# Analysis of post-market adverse events of istradefylline: a real-world study base on FAERS database

**DOI:** 10.1038/s41598-024-58460-6

**Published:** 2024-04-01

**Authors:** Ying Jiang, Rongrong Lu, Qin Zhou, Yuan Shen, Haohao Zhu

**Affiliations:** Mental Health Center of Jiangnan University, Wuxi Central Rehabilitation Hospital, Wuxi, 214151 Jiangsu China

**Keywords:** Istradefylline, FAERS, Real-world data analysis, Adverse events, Adverse drug reaction, Psychiatric disorders, Therapeutics

## Abstract

Analyze the adverse event (AE) signals of istradefylline based on the FAERS database. By extracting large-scale data from the FAERS database, this study used various signal quantification techniques such as ROR, PRR, BCPNN, and MGPS to calculate and evaluate the ratio and association between istradefylline and specific AEs. In the FAERS database, this study extracted data from the third quarter of 2019 to the first quarter of 2023, totaling 6,749,750 AE reports. After data cleansing and drug screening, a total of 3633 AE reports related to istradefylline were included for analysis. Based on four calculation methods, this study unearthed 25 System Organ Class (SOC) AE signals and 82 potential preferred terms (PTs) related to istradefylline. The analysis revealed new AEs during istradefylline treatment, including reports of Parkinsonism hyperpyrexia syndrome (n = 3, ROR 178.70, PRR 178.63, IC 1.97, EBGM 165.63), Compulsions (n = 5, ROR 130.12, PRR 130.04, IC 2.53, EBGM 123.02), Deep brain stimulation (n = 10, ROR 114.42, PRR 114.27, IC 3.33, EBGM 108.83), and Freezing phenomenon (n = 60, ROR 97.52, PRR 96.76, IC 5.21, EBGM 92.83). This study provides new risk signals and important insights into the use of istradefylline, but further research and validation are needed, especially for those AE that may occur in actual usage scenarios but are not yet explicitly described in the instructions.

## Introduction

With the advancement of medicine and the discovery of new drugs, the methods and strategies for treating diseases are continually evolving. Parkinson's disease, a neurodegenerative disorder affecting the central nervous system, has long been a focus of research in terms of treatment strategies and drug selection. In the course of treating Parkinson's disease, Levodopa (L-dopa) has long been considered the most effective treatment drug. With the progression of the disease and long-term use of drugs like Levodopa, many patients begin to experience what is called an “off period,” where the drug effect diminishes or disappears, leading to a significant worsening of symptoms. During this time, patients may experience pronounced motor disturbances, such as increased tremors, rigidity, and slowed movement^1–3^. The global prevalence of Parkinson's disease is around 0.3%, rising to 1% in the population over 65 years old. After several years of Levodopa treatment, it is estimated that 40–50% of Parkinson's patients will experience an off period^4^. This not only severely impacts patients' daily lives and quality of life but may also lead to anxiety, depression, and other psychological issues.

Nourianz®, an orally administered Parkinson's disease medication developed by Kyowa Kirin Co., Ltd. of Japan, received formal approval from the U.S. FDA on August 27, 2019, with the primary active ingredient being istradefylline^5^. Istradefylline, as an adenosine receptor antagonist, has demonstrated potential therapeutic value in Parkinson's disease treatment, especially as an adjunct to Levodopa/Carbidopa treatment^6,7^. However, as istradefylline is a newly marketed drug, its widespread use is accompanied by concerns and attention regarding potential adverse reactions^8,9^. Continuous monitoring and assessment of the safety of new drugs are especially crucial to better provide treatment recommendations to patients.

FAERS (FDA Adverse Event Reporting System) is a database storing numerous reports related to drug adverse events (AEs), providing a rich source of data for research on drug adverse reactions^10,11^. By conducting an in-depth analysis of these data, this study can better assess the safety of istradefylline in actual clinical applications. In this context, this study aims to utilize the FAERS database and employ various signal quantification techniques to conduct a comprehensive analysis of istradefylline's AE signals, thereby providing more empirical data support for clinical decision-making.

## Materials and methods

### Study design and data source

This study conducted a retrospective pharmacovigilance research based on the FAERS database to investigate the association between istradefylline and potential AEs. FAERS is an essential database of the U.S. Food and Drug Administration, collecting information on AEs reported by patients and healthcare professionals during drug use. This study extracted data from the FAERS database from the third quarter of 2019 to the first quarter of 2023 to obtain the latest information on drug AEs. To ensure the specificity and accuracy of the research, this study restricted the search to “istradefylline” and “Nourianz” as the primary suspected drugs. By using software tools (SAS) for MySQL, data were collected, preprocessed, and cleaned to ensure accuracy and completeness^12^.

**Figure 1:**
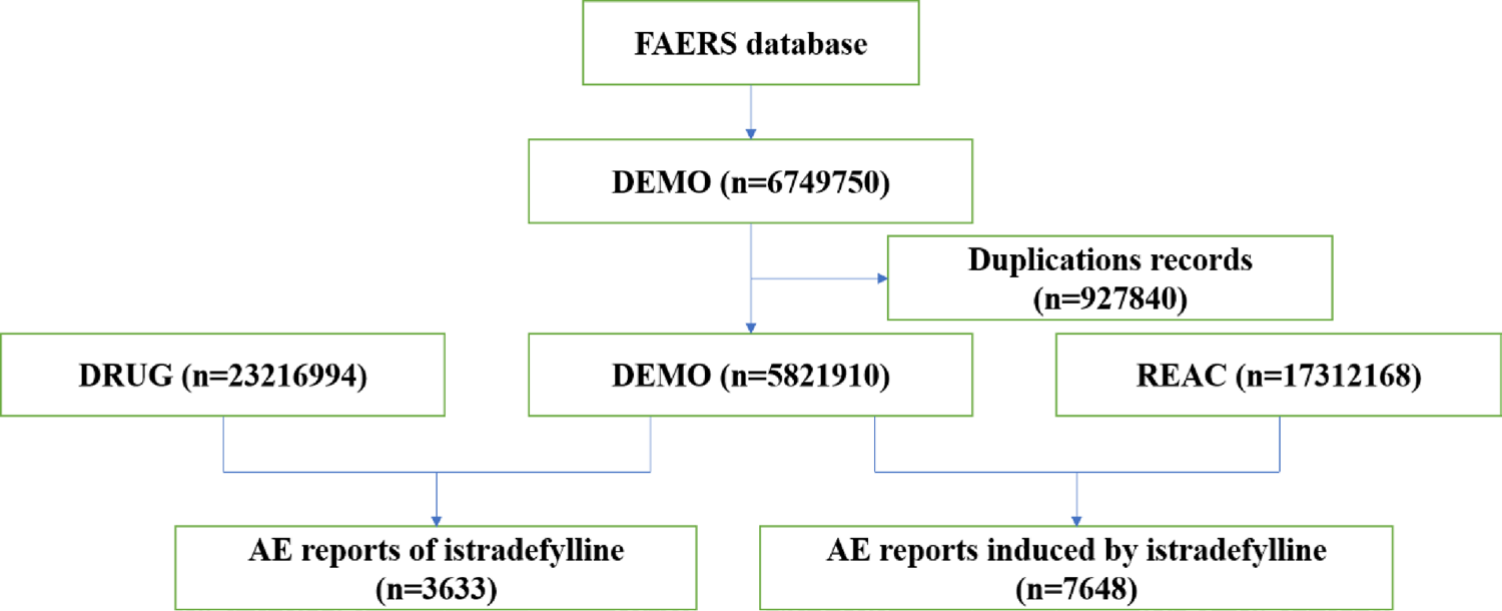
The flow chart of the data analysis.

Following the FDA-recommended method for removing duplicate reports, we select the PRIMARYID, CASEID, and FDA_DT fields from the DEMO table. We sort by CASEID, FDA_DT, and then PRIMARYID. For reports with the same CASEID, we retain the one with the largest FDA_DT value. Secondly, for reports where both CASEID and FDA_DT are the same, we retain the one with the largest PRIMARYID value. Since the first quarter of 2019, each quarterly data package has included a list of deleted reports. After data deduplication, we remove reports based on the CASEID listed in the deleted reports list. The details could be found in Fig. 1.

Subsequently, the data were mapped to RxNorm and MedDRA concept libraries. Additionally, for the preferred terms (PT), we carried out standardization and translation, ensuring the consistency of the data. To present the data, this study merged AE reports with the same PTs. Simultaneously, using the System Organ Class (SOC) method, PTs were categorized and organized to better summarize and analyze the characteristics of AEs.

### Statistical analysis

This study employed various statistical analysis methods to evaluate the association between istradefylline and potential AEs. The calculation methods are shown in Tables 1 and 2, starting with a descriptive statistical analysis of the AE data related to istradefylline. Then, using disproportionality methods including Relative Odds Ratio (ROR)^13^, Proportional Reporting Ratio (PRR)^14^, Bayesian Confidence Propagation Neural Network (BCPNN)^15^, and Multi-item Gamma Poisson Shrinker (MGPS) algorithms^16^, possible associations and anomalies were detected, aiding in determining whether the AEs related to istradefylline have statistical significance.

**Table 1:** Four grid table.

	Target AEs	Non-target AEs	Total
Istradefylline	a	b	a + b
Non-istradefylline	c	d	c + d
Total	a + c	b + d	N = a + b + c + d

Equation: a, number of reports containing both the target drug and target adverse drug reaction; b, number of reports containing other adverse drug reaction of the target drug; c, number of reports containing the target adverse drug reaction of other drugs; d, number of reports containing other drugs and other adverse drug reactions.

**Table 2:** ROR, PRR, BCPNN, and EBGM methods, formulas, and thresholds.

Method	Formula	Threshold
ROR	${\text{ROR}} = \frac{{\left( {a/c} \right)}}{{\left( {b/d} \right)}} = \frac{ad}{{bc}}$ ${\text{SE}}\left( {{\text{lnROR}}} \right) = \sqrt {\left( {\frac{1}{{\text{a}}} + \frac{1}{{\text{b}}} + \frac{1}{{\text{c}}} + \frac{1}{{\text{d}}}} \right)}$ $95{\text{\% CI}} = {\text{e}}^{{{\text{ln}}\left( {{\text{ROR}}} \right) \pm 1.96\sqrt {{ }\left( {\frac{1}{{\text{a}}} + \frac{1}{{\text{b}}} + \frac{1}{{\text{c}}} + \frac{1}{{\text{d}}}} \right)} }}$	a ≥ 3 and 95% CI (lower limit) > 1
PRR	${\text{PRR = }}\frac{{a/\left( {a + b} \right)}}{{c/\left( {c + d} \right)}}$ ${\text{SE}}\left( {{\text{lnPRR}}} \right) = \sqrt {\frac{1}{a} - \frac{1}{a + b} + \frac{1}{c} - \frac{1}{{{\text{c}} + {\text{d}}}}}$ ${95}\% {\text{CI}}\, = \,e^{{{\text{ln}}\left( {{\text{PRR}}} \right) \pm 1.96\sqrt { \frac{1}{a} - \frac{1}{a + b} + \frac{1}{c} - \frac{1}{{{\text{c}} + {\text{d}}}}} }}$	a ≥ 3 and 95% CI (lower limit) > 1
BCPNN	IC = $$log_{2} \frac{{p\left( {x,y} \right)}}{p\left( x \right)p\left( y \right)} = log_{2} \frac{{a\left( {a + b + c + d} \right)}}{{\left( {a + b} \right)\left( {a + c} \right)}}$$ E(IC) = $$log_{2} \frac{{\left( {a + \gamma 11} \right)\left( {a + b + c + d + \alpha } \right)\left( {a + b + c + d + \beta } \right)}}{{\left( { + b + c + d + \gamma } \right)\left( {a + b + \alpha 1} \right)\left( {a + c + \beta 1} \right)}}$$ V(IC) = $$\frac{1}{{\left( {ln2} \right)^{2} }}\left\{ {\left[ {\frac{{\left( {a + b + c + d} \right) - a + \gamma - \gamma 11}}{{\left( {a + \gamma 11} \right)\left( {1 + a + b + c + d + \gamma } \right)}}} \right] + \left[ {\frac{{\left( {a + b + c + d} \right) - \left( {a + b} \right) + \alpha - \alpha 1}}{{\left( {a + b + \alpha 1} \right)\left( {1 + a + b + c + d + \alpha } \right)}}} \right] + \left[ {\frac{{\left( {a + b + c + d} \right) - \left( {a + c} \right) + \beta - \beta 1}}{{\left( {a + c + \beta 1} \right)\left( {1 + a + b + c + d + \beta } \right)}}} \right]} \right\}$$$$\gamma = \gamma 11\frac{{\left( {a + b + c + d + \alpha } \right)\left( {a + b + c + d + \beta } \right)}}{{\left( {a + b + \alpha 1} \right)\left( {a + c + \beta 1} \right)}}$$ *IC-2SD* = *E(IC)-2* $$\sqrt {V\left( {IC} \right)}$$	IC025 > 0
EBGM	${\text{EBGM}} = \frac{{a\left( {a + b + c + d} \right)}}{{\left( {a + c} \right)\left( {a + b} \right)}}$ $95{\text{\% CI}} = {\text{e}}^{{{\text{ln}}\left( {{\text{EBGM}}} \right) \pm 1.96\sqrt {{ }\left( {\frac{1}{{\text{a}}} + \frac{1}{{\text{b}}} + \frac{1}{{\text{c}}} + \frac{1}{{\text{d}}}} \right)} }}$	EBGM05 > 2

*95% CI* 95% confidence interval, *N* the number of reports, *χ2* chi-squared, *IC* information component, *IC025* the lower limit of 95% CI of the IC, *E(IC)* the IC expectations, *V(IC)* the variance of IC, *EBGM* empirical Bayesian geometric mean, *EBGM05* the lower limit of 95% CI of EBGM.

## Results

### Basic information on AE reports

This study extracted data from the FAERS database from the third quarter of 2019 to the first quarter of 2023, with a total of 6,749,750 AE reports. After data cleaning and drug screening, this study included a total of 3633 AE reports related to istradefylline for analysis. From Table 3, it is observed that the majority of AE reports (98.51%) did not provide gender information, and the gender distribution cannot be determined from the known gender reports, even though 98.54% of the reports did not involve the patient's specific age. However, in the part with age information provided, the patient group aged 75 and above was most often reported, with a proportion of 0.85%. Pharmacists were the main source of reports, providing the majority, at 87.75%. The vast majority of AE reports (98.76%) came from the United States, while reports from Japan accounted for 0.88%. Excluding the data for the first quarter of 2023, AE reports in 2020 were the highest, accounting for 37.88% of total reports, indicating a possible increase in istradefylline use or strengthened monitoring in that year. In clinical outcomes, other than unspecified serious AEs, the mortality rate accounted for 10.68% of all reports, indicating the presence of certain serious AEs associated with this drug. Initial or extended hospitalization reports accounted for 8.23%, further emphasizing the drug's potential severity. Excluding data missing or outliers associated with the time of AE occurrence, the first 7 days after the start of medication were the most common period for AEs, accounting for 6.58%.

**Table 3:** Basic information on AE reports for istradefylline.

Factors	Number of events (%)
Gender	
Female	18 (0.50)
Male	36 (0.99)
Unknown	3579 (98.51)
Age	
< 18	1 (0.03)
≥ 18, < 45	1 (0.03)
≥ 45, < 65	2 (0.06)
≥ 65, < 75	18 (0.50)
≥ 75	31 (0.85)
Unknown	3580 (98.54)
Reporter	
Pharmacist	3188 (87.75)
Consumer	305 (8.40)
Physician	60 (1.65)
Other health professionals	70 (1.93)
Unknown	10 (0.28)
Reported countries	
United States	3588 (98.76)
Not specified	12 (0.33)
Germany	1 (0.03)
Japan	32 (0.88)
Report year	
2019	80 (2.20)
2020	1376 (37.88)
2021	1071 (29.48)
2022	920 (25.32)
2023	186 (5.12)
Serious outcomes	
Death	388 (10.68)
Disability	11 (0.30)
Hospitalization—initial or prolonged	299 (8.23)
Life-threatening	8 (0.22)
AE occurrence time—medication date (days)	
0–7	239 (6.58)
7–28	91 (2.50)
28–60	87 (2.39)
≥ 60	481 (13.24)
Unknown	2735 (75.28)

### Results of risk signal mining

Based on four computational methods, this study mined 25 AE signals related to istradefylline in SOCs, as detailed in Table 4. Using the most stringent EBGM method to rank the AE signals, the five most prominent categories were: Surgical and medical procedures (n = 267, ROR 4.03, PRR 3.92, IC 1.95, EBGM 3.92), Nervous system disorders (n = 1567, ROR 4.17, PRR 3.52, IC 1.81, EBGM 3.52), Psychiatric disorders (n = 1182, ROR 3.85, PRR 3.41, IC 1.76, EBGM 3.40), Endocrine disorders (n = 2, ROR 3.16, PRR 3.16, IC 0.88, EBGM 3.16), and General disorders and administration site conditions (n = 2147, ROR 2.25, PRR 1.90, IC 0.92, EBGM 1.90). Based on the number of reports, the most commonly reported SOCs were: General disorders and administration site conditions, Nervous system disorders, Psychiatric disorders, Injury, poisoning and procedural complications, and Gastrointestinal disorders. After comparing with the drug leaflet, this study identified Surgical and medical procedures, Endocrine disorders, General disorders and administration site conditions, Reproductive system and breast disorders, and Injury, poisoning and procedural complications as new potential AEs related to istradefylline use that are not yet mentioned in the leaflet and have significant signal strength. This prompts us to further focus and evaluate these SOCs' AEs for istradefylline use. The calculation details could be found in Supplementary Table S1.

**Table 4:** Istradefylline AE signals and affected SOCs.

SSOCs	SOC code	Case reports	ROR (95% CI)	PRR (95% CI)	χ2	IC (IC025)	EBGM (EBGM05)
Surgical and medical procedures	10042613	267	4.03 (3.56–4.55)	3.92 (3.49–4.41)	585.5756	1.95 (1.77)	3.92 (3.47)
Nervous system disorders	10029205	1567	4.17 (3.95–4.41)	3.52 (3.37–3.68)	2998.62	1.81 (1.73)	3.52 (3.33)
Psychiatric disorders	10037175	1182	3.85 (3.62–4.09)	3.41 (3.23–3.59)	2103.496	1.76 (1.67)	3.40 (3.20)
Endocrine disorders	10014698	2	3.16 (0.79–12.65)	3.16 (0.79–12.65)	2.949189	0.88 (− 0.79)	3.16 (0.79)
General disorders and administration site conditions	10018065	2147	2.25 (2.14–2.36)	1.90 (1.83–1.97)	1067.905	0.92 (0.85)	1.90 (1.80)
Gastrointestinal disorders	10017947	637	1.37 (1.26–1.48)	1.34 (1.24–1.44)	57.87756	0.42 (0.30)	1.34 (1.23)
Reproductive system and breast disorders	10038604	5	1.29 (0.54–3.10)	1.29 (0.54–3.09)	0.320649	0.30 (− 0.88)	1.29 (0.54)
Injury, poisoning and procedural complications	10022117	710	1.18 (1.10–1.28)	1.17 (1.09–1.25)	18.33846	0.22 (0.11)	1.17 (1.08)
Eye disorders	10015919	61	1.14 (0.89–1.47)	1.14 (0.89–1.46)	1.067662	0.19 (− 0.18)	1.14 (0.89)
Ear and labyrinth disorders	10013993	22	1.10 (0.73–1.68)	1.10 (0.73–1.68)	0.214104	0.14 (− 0.47)	1.10 (0.73)
Musculoskeletal and connective tissue disorders	10028395	255	0.98 (0.87–1.11)	0.98 (0.87–1.11)	0.069399	− 0.02 (− 0.21)	0.98 (0.87)
Social circumstances	10041244	20	0.91 (0.59–1.41)	0.91 (0.59–1.41)	0.169956	− 0.13 (− 0.76)	0.91 (0.59)
Vascular disorders	10047065	79	0.79 (0.63–0.98)	0.79 (0.63–0.98)	4.52041	− 0.34 (− 0.66)	0.79 (0.63)
Metabolism and nutrition disorders	10027433	65	0.76 (0.59–0.97)	0.76 (0.60–0.97)	5.003891	− 0.39 (− 0.75)	0.76 (0.60)
Product issues	10077536	4	0.74 (0.28–1.96)	0.74 (0.28–1.96)	0.376328	− 0.36 (− 1.65)	0.74 (0.28)
Renal and urinary disorders	10038359	46	0.67 (0.50–0.89)	0.67 (0.50–0.90)	7.487107	− 0.57 (− 0.99)	0.67 (0.50)
Cardiac disorders	10007541	50	0.53 (0.40–0.70)	0.53 (0.40–0.70)	20.63439	− 0.89 (− 1.30)	0.53 (0.40)
Skin and subcutaneous tissue disorders	10040785	150	0.51 (0.43–0.60)	0.52 (0.44–0.61)	69.83	− 0.94 (− 1.18)	0.52 (0.44)
Investigations	10022891	110	0.51 (0.42–0.62)	0.52 (0.43–0.62)	50.82818	− 0.94 (− 1.22)	0.52 (0.43)
Infections and infestations	10021881	134	0.50 (0.42–0.60)	0.51 (0.43–0.60)	65.03	− 0.96 (− 1.21)	0.51 (0.43)
Respiratory, thoracic and mediastinal disorders	10038738	89	0.39 (0.32–0.48)	0.40 (0.32–0.49)	84.14573	− 1.32 (− 1.63)	0.40 (0.32)
Hepatobiliary disorders	10019805	3	0.30 (0.10–0.93)	0.30 (0.10–0.93)	4.926641	− 1.46 (− 2.91)	0.30 (0.10)
Immune system disorders	10021428	14	0.29 (0.17–0.49)	0.29 (0.17–0.49)	24.66353	− 1.72 (− 2.47)	0.29 (0.17)
Blood and lymphatic system disorders	10005329	6	0.28 (0.13–0.63)	0.28 (0.13–0.63)	10.98643	− 1.67 (− 2.76)	0.28 (0.13)
Neoplasms benign, malignant and unspecified (incl cysts and polyps)	10029104	23	0.25 (0.17–0.38)	0.26 (0.17–0.38)	50.55851	− 1.92 (− 2.51)	0.26 (0.17)

Furthermore, this study identified 82 potential PTs. Among these PTs, based on EBGM calculations, Table 5 lists the top 30 PTs by strength. Although Parkinsonism hyperpyrexia syndrome, Emergency care, Compulsions, Deep brain stimulation, and Freezing phenomenon were reported relatively infrequently, they ranked in the top five in AE signal strength, and these AEs were not mentioned in the drug leaflet, showing their value as new potential AEs. Dyskinesia and Hallucination were among the more commonly reported AEs, aligning with the descriptions found in the medication leaflet. Of particular note, although Therapy non-responder and Parkinson's disease were not recorded in the leaflet, their frequency of occurrence was also relatively high, and clinical attention should be paid. The calculation details could be found in Supplementary Table S2.

**Table 5:** The top 30 signal strength of AEs of Istradefylline ranked by EBGM at the PTs level.

SOC	PTs	Case reports	ROR (95% CI)	PRR (95% CI)	χ2	IC (IC025)	EBGM (EBGM05)
Nervous system disorders	Parkinsonism hyperpyrexia syndrome	3	178.70 (55.15–579.02)	178.63 (55.15–578.55)	491.1285	1.97 (0.46)	165.63 (51.12)
Surgical and medical procedures	Emergency care	68	140.48 (109.85–179.64)	139.24 (109.11–177.69)	8792.255	5.51 (5.15)	131.22 (102.62)
Psychiatric disorders	Compulsions	5	130.12 (52.82–320.57)	130.04 (52.81–320.19)	605.4255	2.53 (1.31)	123.02 (49.93)
Surgical and medical procedures	Deep brain stimulation	10	114.42 (60.60–216.06)	114.27 (60.57–215.61)	1068.856	3.33 (2.44)	108.83 (57.63)
Nervous system disorders	Freezing phenomenon	60	97.52 (75.24–126.40)	96.76 (74.80–125.17)	5453.6	5.21 (4.83)	92.83 (71.63)
Nervous system disorders	Dyskinesia^a^	316	79.71 (71.10–89.38)	76.46 (68.51–85.34)	22,777.28	5.91 (5.74)	73.99 (65.99)
Psychiatric disorders	Sleep talking^a^	9	68.88 (35.47–133.74)	68.80 (35.46–133.48)	583.561	3.14 (2.21)	66.80 (34.40)
Nervous system disorders	On and off phenomenon	47	56.58 (42.32–75.63)	56.24 (42.14–75.05)	2488.387	4.69 (4.27)	54.90 (41.07)
Psychiatric disorders	Impulse-control disorder^a^	11	55.76 (30.64–101.46)	55.68 (30.63–101.23)	576.5029	3.32 (2.47)	54.37 (29.88)
Psychiatric disorders	Libido increased	8	48.19 (23.91–97.11)	48.14 (23.91–96.94)	361.6075	2.94 (1.97)	47.16 (23.40)
General disorders and administration site conditions	General symptom	17	46.84 (28.96–75.75)	46.74 (28.93–75.51)	745.5298	3.71 (3.03)	45.81 (28.33)
Psychiatric disorders	Fear of falling^a^	4	43.53 (16.18–117.12)	43.51 (16.18–117.00)	163.0069	2.19 (0.89)	42.71 (15.88)
Psychiatric disorders	Hallucination^a^	313	39.81 (35.52–44.62)	38.22 (34.26–42.64)	11,168.48	5.07 (4.91)	37.60 (33.55)
General disorders and administration site conditions	Therapy non-responder	250	35.40 (31.18–40.20)	34.28 (30.32–38.76)	7964.462	4.90 (4.71)	33.78 (29.76)
Nervous system disorders	Parkinson's disease	92	34.05 (27.68–41.88)	33.65 (27.42–41.29)	2872.897	4.62 (4.32)	33.17 (26.97)
Psychiatric disorders	Abnormal sleep-related event^a^	3	31.58 (10.10–98.72)	31.57 (10.10–98.64)	87.58832	1.87 (0.41)	31.15 (9.97)
Injury, poisoning and procedural complications	Product dispensing issue	11	26.54 (14.64–48.11)	26.51 (14.63–48.01)	266.866	3.08 (2.24)	26.21 (14.46)
General disorders and administration site conditions	Inhibitory drug interaction^a^	4	25.58 (9.54–68.55)	25.57 (9.54–68.48)	93.36872	2.11 (0.81)	25.29 (9.44)
General disorders and administration site conditions	Energy increased^a^	15	23.55 (14.15–39.18)	23.50 (14.14–39.07)	319.8975	3.28 (2.56)	23.27 (13.99)
Psychiatric disorders	Impulsive behaviour^a^	7	23.42 (11.12–49.32)	23.39 (11.11–49.25)	148.5336	2.62 (1.59)	23.17 (11.00)
Nervous system disorders	Head titubation^a^	3	22.05 (7.07–68.75)	22.04 (7.07–68.69)	59.67152	1.81 (0.36)	21.84 (7.00)
Metabolism and nutrition disorders	Food refusal^a^	3	21.83 (7.00–68.08)	21.83 (7.00–68.03)	59.04671	1.81 (0.36)	21.63 (6.94)
Psychiatric disorders	Obsessive thoughts	3	19.24 (6.17–59.94)	19.23 (6.17–59.90)	51.40739	1.79 (0.34)	19.08 (6.12)
Psychiatric disorders	Hallucination, visual^a^	39	15.93 (11.61–21.84)	15.85 (11.58–21.70)	539.0466	3.52 (3.06)	15.75 (11.48)
Injury, poisoning and procedural complications	Product dispensing error	75	15.82 (12.59–19.88)	15.68 (12.51–19.65)	1024.172	3.71 (3.37)	15.58 (12.40)
Psychiatric disorders	Somnambulism	8	15.61 (7.78–31.29)	15.59 (7.78–31.24)	108.4996	2.57 (1.60)	15.49 (7.73)
Nervous system disorders	Bradykinesia	13	13.82 (8.01–23.85)	13.80 (8.00–23.79)	153.3802	2.85 (2.07)	13.72 (7.95)
Psychiatric disorders	Obsessive–compulsive disorder^a^	10	13.52 (7.26–25.19)	13.51 (7.26–25.14)	115.1459	2.66 (1.78)	13.43 (7.21)
Psychiatric disorders	Gambling disorder	3	12.19 (3.92–37.92)	12.19 (3.92–37.89)	30.64153	1.68 (0.23)	12.13 (3.90)
General disorders and administration site conditions	Adverse reaction	18	12.20 (7.67–19.40)	12.17 (7.66–19.33)	183.621	2.93 (2.27)	12.11 (7.62)

^a^Represents that the PT is present in the drug leaflet.

## Discussions

Parkinson's disease is a progressive neurodegenerative disease, and in the past decade, mechanisms that regulate basal ganglia motor pathways without affecting dopamine levels have become the target for the development of antitremor ma huang drugs. Although adamantane and anticholinergic drugs are used clinically to control motor symptoms, their application is limited due to limited efficacy and potential side effects. Meanwhile, atypical antipsychotic drugs, anti-anxiety, anti-depressants, and cholinesterase inhibitors are mainly used to manage non-motor symptoms of Parkinson's disease, but they are also relatively limited in treating core symptoms^17^. To date, only istradefylline has successfully passed phase III clinical trials and has been approved for clinical treatment of Parkinson's disease^18,19^. Although drugs are subjected to rigorous clinical trials before going on the market, adverse reactions that may occur in actual applications are not always completely predictable. Continuous research and monitoring of istradefylline can help identify and respond to these adverse reactions in a timely manner. This study, based on the FAERS database, conducted AE signal mining and evaluation for istradefylline. Particularly, those AEs not explicitly mentioned in the official drug instructions provide important information for future drug safety supervision and clinical practice.

This study extracted a total of 6,749,750 AE reports from the FAERS database from the third quarter of 2019 to the first quarter of 2023, including 3633 AE reports related to istradefylline. Through data cleaning and drug screening, we obtained rich information about istradefylline. However, we noticed that there was a significant absence of gender and age information in the reports, and we need to be cautious when analyzing these features. From the reports with known genders, there were no clear gender distribution characteristics. Although age information is missing from most reports, among those providing age information, the patient group aged 75 and over was the most common. Pharmacists were the main source of reporting, occupying a considerable proportion. Additionally, the vast majority of reports came from the United States, with a certain percentage from Japan. These data reveal the basic characteristics of istradefylline AE reporting and provide a background for further analysis.

This study found that some AEs related to the use of istradefylline, such as Surgical and medical procedures, Endocrine disorders, General disorders and administration site conditions, were not mentioned in the drug instructions. This indicates that the drug instructions may need to be further refined to include more comprehensive information on AEs. At the same time, some frequently occurring AEs like Dyskinesia and Hallucination are consistent with the records in the instructions, emphasizing the importance of these known risks. Although some AE cases are relatively few, such as Parkinsonism hyperpyrexia syndrome, Emergency care, Compulsions, Deep brain stimulation, and Freezing phenomenon, they rank among the top five in signal strength, suggesting these new, rare but potentially significant AEs. It is especially noteworthy that although Therapy non-responder and Parkinson's disease were not included in the instructions, due to their relatively high frequency of reports, sufficient attention should still be given in actual clinical practice.

Based on the study results, this research focuses on new AEs related to istradefylline, including Parkinsonism hyperpyrexia syndrome, Compulsions, Deep brain stimulation, and Freezing phenomenon, but to understand their potential connection and mechanism with istradefylline, further exploration is needed in this study.

Parkinsonism hyperpyrexia syndrome (PHS) is a rare but serious reaction^20^. Istradefylline is an antagonist of adenosine A2a receptors, which often coexist with dopamine D2 receptors on many neurons. In Parkinson's disease models, the activation of adenosine A2a receptors is opposed to the inhibitory effect of D2 receptors. Therefore, by antagonizing adenosine A2a receptors, istradefylline can enhance the activity of D2 receptors. However, this interaction may lead to overactivation of the dopamine system in some cases, triggering PHS^21^. Simultaneously, adenosine A2a receptors are more common in the striatum, a key part of the basal ganglia, related to motor control, emotion, and autonomic nervous function regulation^22^. Istradefylline may induce changes in the signaling pathways within the basal ganglia, thus causing symptoms of PHS^7^.

Compulsions induced by drugs have been widely reported in drugs related to dopamine action, especially in Parkinson's disease patients using dopamine receptor agonists. Given istradefylline's dopamine-enhancing effect, the risk of impulsive behavior may increase^23^. Therefore, physicians and patients need to remain vigilant for possible behavioral changes. Deep brain stimulation (DBS) is a surgical method for treating Parkinson's disease, regulating abnormal neural electrical activity by implanting electrodes in the brain^24^. Since istradefylline is approved as an add-on therapy to treat off-episodes of levodopa. Therefore, patients receiving this medication may have insufficient response to levodopa and are likely candidates for procedures such as DBS.

Freezing phenomenon is a common symptom in Parkinson's disease, characterized by the patient suddenly stopping moving, especially when starting to move or turning. The occurrence of this symptom is related to the imbalance of dopamine and other neurotransmitters in the brain^25^. Since adenosine A2a receptors and dopamine D2 receptors often coexist on the same neuron, and their actions are often antagonistic, the antagonistic effect of istradefylline may enhance the effect of dopamine. While this may be beneficial in alleviating some symptoms of Parkinson's disease, it might also cause or exacerbate freezing phenomena. Also, the changes that istradefylline makes to the activity of striatal neurons may affect neural pathways related to freezing^26,27^.

The AE discussed above reveal possible risks of istradefylline in the treatment of Parkinson's disease. However, the study has some limitations. First, the AE reports recorded in the FAERS database are spontaneous in nature, so there may be reporting bias. Although this study utilized various computational methods for signal mining, these methods have their inherent limitations and need to be evaluated in conjunction with actual clinical situations. Moreover, it is worth noting that although this study identified new potential AEs associated with the use of istradefylline, it does not mean that these events are entirely caused by the drug. They may be influenced by various factors, including patient baseline characteristics, comorbid diseases, and concomitant medications. This study only analyzed reports related to istradefylline and did not set other drugs as control groups. Therefore, it is unable to conduct sensitivity analyses specific to Parkinson’s disease medications, making it challenging to eliminate the confounding factor of Parkinson’s disease symptoms themselves being reported as AEs in the spontaneous reports. Further research is necessary to establish the causal relationship between these AEs and istradefylline.

## Conclusion

In summary, the study identified new potential AEs related to the use of istradefylline, events not yet mentioned in the instructions and with significant signal strength. These include five prominent categories, such as “Surgical and medical procedures” and “Endocrine disorders,” as well as PTs ranked in the top 30 for intensity, such as “Parkinsonism hyperpyrexia syndrome” and “Emergency care”. However, this is only a preliminary analysis, and further in-depth research and validation are needed to ascertain the clinical significance and authenticity of these risks. In addition, the drug's AEs may be influenced by various factors, including the patient's baseline characteristics, the route of administration, etc. These factors need to be considered in future research. Overall, the results of this study can provide guidance for drug monitoring and safety assessment in clinical practice and offer a basis for appropriate management strategies by drug regulatory agencies and medical institutions.

### Supplementary Information

Supplementary Table S1.

Supplementary Table S2.

## Data Availability

The dataset generated during and analyzed during the current study are available from the corresponding author on reasonable request.
